# DANDRUFF: THE MOST COMMERCIALLY EXPLOITED SKIN DISEASE

**DOI:** 10.4103/0019-5154.62734

**Published:** 2010

**Authors:** S Ranganathan, T Mukhopadhyay

**Affiliations:** *From the CavinKare Research Centre, No.12 Poonamallee Road, Ekkattuthangal, Chennai – 600 097, India.*

**Keywords:** *Dandruff*, *cosmetic problem*, *scalp disease*, *shampoos*

## Abstract

The article discuss in detail about the prevalence, pathophysiology, clinical manifestations of dandruff including the etio-pathology. The article also discusses in detail about various treatment methods available for dandruff. The status of dandruff being amphibious – a disease/disorder, and relatively less medical intervention is sought after for the treatment, dandruff is the most commercially exploited skin and scalp disorder/disease by personal care industries.

## Introduction

Dandruff is a common scalp disorder affecting almost half of the population at the pre-pubertal age and of any gender and ethnicity.[1] No population in any geographical region would have passed through freely without being affected by dandruff at some stage in their life.[2] The word dandruff (dandruff, dandriffe) is of Anglo-Saxon origin, a combination of ‘tan’ meaning ‘tetter’ and ‘drof’ meaning ‘dirty’. Dandruff affects aesthetic value and often causes itching. It has been well established that keratinocytes play a key role in the expression and generation of immunological reactions during dandruff formation.[3] The severity of dandruff may fluctuate with season as it often worsens in winter.[4]

## Pathophysiology

Even today, the debate on whether dandruff has to be treated as a disease or a disorder continues. In the physiological spectrum of scaling, about 487,000cells/sq cm get released normally after detergent treatment and this number goes up to 800,000/sq cm during dandruff and seborrhoeic dermatitis.[5] However, dandruff is non-inflammatory in nature. The real cause for dandruff formation from the normal physiological spectrum of scaling is yet to be understood.

### Dandruff - seborrhoeic dermatitis link

The spectrum of dandruff is difficult to define because it blurs with seborrhoeic dermatitis and some other scaly conditions. The inflammation and extension of scaling outside the scalp exclude the diagnosis of dandruff from seborrhoeic dermatitis.[6] However, many reports suggest a clear link between the two clinical entities - the mildest form of the clinical presentation of seborrhoeic dermatitis as dandruff, where the inflammation is minimal and remain subclinical. Histological examination reveals the scattered presence of lymphoid cells and squirting capillaries in the papillary dermis with hints of spongiosis and focal parakeratosis.[3,7]

Conceptually, dandruff is a dander and represents nothing more than physiologic scaling.[8] Hence it is believed that physiological scaling process requires more of cosmetic management. The response to treatment is commonly swift, but transient. On the contrary, seborrhoeic dermatitis is obviously more inflammatory in nature extending outside the limit of the scalp surface.[5]

### Dandruff composition

Dandruff scale is a cluster of corneocytes, which have retained a large degree of cohesion with one another and detach as such from the surface of the stratum corneum. The size and abundance of scales are heterogeneous from one site to another and over time. Parakeratotic cells often make up part of dandruff. Their numbers are related to the severity of the clinical manifestations, which may also be influenced by seborrhea.[9]

### Microbial etiology of dandruff

There could be several etiopathologic pathways with complex mechanisms, which may cause dandruff. The role of lipophilic yeast belonging to the genus *Malassezia* was widely accepted to play a role in dandruff way back in 1846.[8,10] Eichstedt was the first to recognize the presence of this fungus in the disease pityriasis versicolor.[8,11] The scalp form as biocenose for various organisms such as *Staphylococci* spp., *Propionibacterium* spp., and *Malassezia* spp.,[12,13] The density of these organisms varies from 10^3^ to 10^5^ organisms per mm^2^. During dandruff, the levels of *Malassezia* increase by 1.5 to 2 times its normal level. It has been debated that the quantitative microbial assessment of all kinds does not indicate the role of yeast; the abundance that might have been proportional to the volume of scales which it colonizes or be responsible for the altered desquamation. Interestingly, the specific relationship between a species of *Malassezia* appears to be the strategy for treating dandruff.

In response to the use of antifungal preparations, population of *Malassezia* reduces, but the bacterial population is seldom affected. After withdrawal of treatment, the clinical situation recurs and *Malassezia* population increases to the initial level. Another interesting aspect on the microbial cause of dandruff is the positive treatment response of dandruff to various steroids. Steroids are known to suppress the immune flare-up and the microbe will advantageously utilize the immune suppressed opportunity. But the good treatment response and prolonged remission period with steroid treatment raises a doubt on the microbial cause of dandruff. Whether the increased *Malassezia* count is the result of abundant scales or abundant scales is due to the increased *Malassezia* population lack clear explanation till date.[12] There are seven species of *Malassezia - M. globosa, M. resticta, M. obtuse, M. sloofiae, M. sympodialis, M. furfur* and *M. pachydermatis,* which have been recognized in dandruff formation.[14] However, none of the species of *Malssezia* have fulfilled the Koch's postulates as a pathogen of dandruff till date.

### Role of corneocytes in dandruff

Both in dandruff and seborrhoeic dermatitis, the population of *Malassezia* is not uniform throughout the skin surface and inside the stratum corneum.[1,3] Clumpy adherence of the yeast is seen in some corneocytes, whereas other corneocytes in the neighborhood region harbor few of these yeast. It is presumed that perhaps the *Malassezia* binding site may differ in corneocytes. Another postulate is the natural antifungal peptides of the innate immunity to the above cause. The colonization of the yeast boosts the expression of β–defensin-2 by keratinocytes.[15] In dandruff, their expression could be impaired at some sites where the abundance of *Malassezia* is more. It is known that *Malassezia* has antigenic and pro-inflammatory properties stimulating both innate & acquired immune response and neuro immune sensorial response as well.[16] In dandruff, the immune response is not altered.

The *Malassezia* – corneocyte hypothesis still leaves some doubt. It does not explain why scaling at low grade (2-5 mg/cm scalp/2 days) with low parakeratotic index persist despite dandruff being resolved and *Malassezia* largely removed.[5] It is hypothesized that the antifungal agents may not be able to eradicate deep-seated yeasts allowing a minimal inflammatory reaction to be maintained or the anti inflammatory activity claimed in the antidandruff agents is not effective enough *in vivo.*[17] The adverse reaction such as irritant dermatitis or contact allergic dermatitis may be provoked by the treatment agents also.[18]

### Non-microbial etiopathology of dandruff

The non-microbial cause for dandruff is well established. Excessive exposure to sunlight is known to cause desquamation of the scalp[19,20] Minimal irritation of scalp due to over shampooing, frequent combing, use of certain cosmetic products, dusts and dirt also, to some extent, cause dandruff. However, there is no sufficient experimental evidence to the above assumptions.[3]

## Role of Lipids

Human scalp is very androgen sensitive and sebum rich.[3,21] The sebum forms an ideal nutrient in the biocenose and sebum formation starts with the onset of puberty. However, sebum excretion rate in dandruff infected and non-infected subjects was found to be same. Many subjects with oily scalp did not show dandruff as well.[4] This clearly shows that lipids may facilitate to some extent but not be the primary cause. Many authors presume that host susceptibility factors play a major role in dandruff formation. It is already known that skin surface lipids influence the transient form of *M. orbiculare* and *M. furfur*. It is also known that dandruff affects people at puberty and middle age more than elderly subjects.[8] *In vitro* findings suggest that cholesterol and cholesterol esters induce hyphal formation in *Malassezia*. However, the quantitative or qualitative difference in skin lipids in pityriasis infected and non-infected subjects doesn't seem to vary very significantly.

Many authors have considered that host susceptibility factors account more for dandruff than the lipase activity of the microbe. Although there is sufficient evidence on the growth promotion role of various lipids on *Malassezia*, a clear-cut correlation on the quantitative or qualitative profile of lipids in the infected and control subjects is lacking.[22] An *in vitro* study using Tween 80, a water soluble ester of low chain (C17) fatty acids, which can serve as substrate both for lipase and esterase reveal that growth inhibition was observed with the addition of esterase inhibitors such as di-isopropyl fluorophosphates or quinine while the lipase inhibitors sodium fluoride seldom affects the growth. Studies on the growth supporting ability of various water-soluble triglycerides such as glyceryl esters of long chain fatty acids triolein (C17) and short chain tributyrin (C4) showed that long chain fatty acids have growth-supporting ability.[22]

### Dandruff rating

Visual scoring is widely practiced to assess the severity of dandruff. It is always necessary to give two-week washout period prior to start of any clinical trial.[3] The examination of the entire scalp is advisable rather than defined specific area. Dandruff quantification by bioinstrumental methods such as squamometry and photography are also employed.[20,23]

### Dandruff and hair

The severity of dandruff ranges from discrete to severe among subjects, possibly the scales may be trapped in the mesh of crowded terminal hair prohibiting them from being lost. This dandruff-hair relationship may, in part, explain the absence of dandruff in bald pates and hairless or shaved sites and in regions of vellus hairs. The presence of dandruff may precede or accompany telogen effluvium.[24] It may also exacerbate androgenetic alopecia. On a two-day survey, it has been observed that about 100-300 numbers of hairs were shed in dandruff sufferers instead of 50-100 in normal subjects.[3]

In some cases of dandruff, hair shedding may be a result of alterations in the teloptosis process (exogen phase) and hair eclipse phenomenon. Interestingly, some of the antidandruff compounds, especially ketoconazole, may limit the progression of androgenic alopecia.[24–26]

### ABO blood groups and dandruff

Another interesting study linking the role of ABO blood group carriage rate of dandruff reveals that ABO blood group system does not play a role either in the prevalence or chronicity of dandruff.[27] The role of ABO blood group in certain other fungal skin diseases is well established. The cross reactivity between the fungal cell wall protein and the isoantign of ABO blood groups is presumed to be the cause for the selective susceptibility of certain blood group subjects to fungal diseases.[28]

### Changing perspectives

Many attempts have been made to understand the pathogenesis and pathogenecity of dandruff. De Angelis *et al,*[29] give an interesting result - that *M. restricta* and *M. globosa* are the most prevalent in dandruff affected population than *M. furfur*. They have also shown, concomitant with the elimination of these organisms, the remission of dandruff symptoms in all the cases they have studied. They hypothesized that species' specificity and specific targeting is required to combat dandruff. However, an earlier study of Faergemann in 2002[30] showed the presence of *M. restricta* to a very less extent in dandruff affected population.

A further twist in the understanding of the role of *M.restricta* in dandruff came with studies of Sugita *et al.*[31] The genotype analysis of *M. restricta* revealed that only a specific genotype of the organism plays a significant role in atopic dermatitis and dandruff although the prevalence of the organism is universal. Despite the elusive and idiopathic nature of dandruff, it remains a problem for great commercial exploitation.

## Active ingredients used in Antidandruff (Ad) Preparations [Table 1]

**Table 1:** Review of medicated shampoos used in the treatment of dandruff

Generic Name	Trade Name	Active ingredient
Salicylic acid	T-Sal	Salacid 3%
	Baker's P and S	Salacid 2%
	Ionil plus	Salacid 2%
Salicylic acid and sulfur	MG217 tar free Shampoo	Salacid 3%, Sulfur 5%
	Sebulex	Salacid 2%, sulfur 2%
Zinc pyrithione	Head and shoulders	Zinc pyrithione 1%
	Zincon	Zinc pyrithione 1%
	Dandrex	Zinc pyrithione 1%
	Sebulon	Zinc pyrithione 2%
	DHS zinc	Zinc pyrithione 2%
	ZNP bar	Zinc pyrithione 2%
	Theraplex Z	Zinc pyrithione 2%
Tar	Pentrax	Coal tar extract 7%
	T-Gel XS	Solubilized coal tar 4%
	Doak-tar	Solubilized coal tar 3%
	T-gel	Solubilized coal tar 2%
	Ionil T	Coal tar solution 1%
	Zetar	Whole coal tar 1%
	DHS Tar	Coal tar 0.5%
	Tegrin	Coal tar solution 7%
	Polytar	Polytar 4.5%
	Reme T	Coal tar 5%
Selenium sulfide	Selsun blue	Selenium sulfide 1%
	Head and shoulders intensive treatment	Selenium sulfide 1%
	Selenium sulfide 1%	Selenium sulfide 1%
	Selseb	Selenium sulfide 2.3%
	Selsun 2.5%*	Selenium sulfide 2.5%
	Exsel 2.5%*	Selenium sulfide 2.5%
	Selenium sulfide 2.5%*	Selenium sulfide 2.5%
Ketoconazole	Nizoral	Ketoconazole 1%
	Nizoral*	Ketoconazole 2%
Ciclopirox	Loprox*	Ciclopirox
Clobetasol	Clobex*	Clobetasol 0.05%
Combinations	Sebutone	Coal tar 0.5%, salicylic acid 2%, sublimed sulfur 2%
	X-seb T plus	Coal tar 10%, salicylic acid 3%
	Tarsum	Coal tar 10%, salicylic acid 2%

* Available only by prescription

### Keratinolytic agents

The pathogenesis of dandruff involves hyper proliferation of keratinocytes, resulting in deregulation of keratinization. The corneocytes clump together, manifesting as large flakes of skin. Essentially, keratolytic agents such as salicylic acid and sulphur loosen the attachments between the corneocytes and allow them to get washed off.[32]

### Salicylic acid

Salicylic acid is a beta hydroxyl acid keratinolytic agent that is useful in removing scaly hyperkeratotic skin. It decreases cell-to-cell adhesion between corneocytes. This agent is widely used in the AD preparations.

### Sulfur

Sulfur, a non-metallic element has both keratolytic and antimicrobial activity. The keratolytic effect is thought to be mediated by the reaction between the sulfur and the cysteine amino acid in the keratinocytes, whereas the antimicrobial effect may depend on the conversion of sulfur to pentathionic acid by normal flora or keratinocytes.[30–34]

### Regulators of keratinization

The zinc pyrithione (ZPT) heals the scalp by normalizing the epithelial keratinization or sebum production or both. Some studies have shown a significant reduction in the number of yeasts after use of ZPT.[35] A study by Warner *et al,*[36] demonstrates a dramatic reduction of structural abnormalities found in dandruff with the use of ZPT; the population abundance of *Malassezia* decreases, parakeratosis gets eliminated and corneocytes lipid inclusions are diminished.

### Tar

Tar is widely used in the treatment of psoriasis and found to be very effective in dandruff as well.[37] The staining properties, odor and mess in using tar limit its choice. Tar preparation work through antiproliferative and cytostatic effects, although definitive analysis is difficult because of the large number of biologically active components in coal tar. Tar products disperse scales, which may reduce *Malassezia* colonization. In the mouse model, it was found that topical application of tar suppresses epidermal DNA synthesis.[37]

Clinical trial of combination of polytar and ZPT based shampoo in Indian population[38] also show that the combination of the above agents are very safe and effective in controlling dandruff and associated symptoms.

### Steroids

The parakeratotic properties of topical corticosteroids depend on the structure of the agent, the vehicle and the skin onto which it is used. Corticosteroids work via their anti-inflammatory and antiproliferative effects.[38]

## Antimicrobial Agents

### Selenium sulfide

It is believed that selenium sulfide controls dandruff via its anti *Malassezia* effect rather than by its antiproliferative effect.[39] although it has an effect in reducing cell turnover. It has anti-seborrheic properties as well as cytostatic effect on cells of the epidermal and follicular epithelium. The excessive oiliness after use of this agent has been reported in many patients as adverse drug effect.

### Imidazole antifungal agents

Imidazole topical antifungals such as ketoconazole act by blocking the biosynthesis of ergosterol, the primary sterol derivative of the fungal cell membrane. Changes in membrane permeability caused by ergosterol depletion are incompatible with fungal growth and survival.[40]

Ketoconazole is a broad spectrum, antimycotic agent that is active against both *Candida* and *M. furfur*. Of all the imidazoles, ketoconazole has become the leading contender among treatment options because of its effectiveness in treating seborrheic dermatitis as well.[41,42]

### Hydroxypyridones

In contrast to the imidazole antifungals, the hydroxypyridones do not affect sterol biosynthesis; instead they interfere with the active transport of essential macromolecule precursor, cell membrane integrity and the respiratory process of cells.[39] Ciclopirox is widely used as an Ad agent in most preparations.

### Naturopathic agents

There are several naturopathic agents which have been claimed to have antidandruff activity. However, in most cases, these naturopathic agents are used in combination with synthetic agents.[43–45] Studies from India[46–48] have shown that the herbal preparations are as effective as synthetic substances in controlling dandruff both by *in vitro* and *in vivo* studies.
